# 2,2′-{1,1′-[2,2′-Oxalylbis(hydrazin-2-yl-1-yl­idene)]diethyl­idyne}dipyridinium bis­(perchlorate) dihydrate

**DOI:** 10.1107/S1600536810010238

**Published:** 2010-03-24

**Authors:** Goran N. Kaluderović, Rabia O. Mohamad Eshkourfu, Santiago Gómez-Ruiz, Dragana Mitić, Katarina K. Andelković

**Affiliations:** a Department of Chemistry, Institute of Chemistry, Technology and Metallurgy, University of Belgrade, Studentski trg 14, 11000 Belgrade, Serbia; b Faculty of Chemistry, University of Belgrade, Studentski trg 16, 11000 Belgrade, Serbia; c Departamento de Química Inorgánica y Analìtica, E.S.C.E.T., Universidad Rey Juan Carlos, 28933 Móstoles, Madrid, Spain

## Abstract

The title salt, C_16_H_18_N_6_O_2_^2+^·2ClO_4_^−^·2H_2_O, was obtained unintentionally as a major product in the reaction of Zn(ClO_4_)_2_·6H_2_O with the *N*′,*N*′^2^-bis­[(1*E*)-1-(2-pyrid­yl)ethyl­idene]ethanedihydrazide (H_2_ *L*) ligand. The (H_4_ *L*)^2+^ cation lies across a centre of inversion. The pyridiniumimine fragments of (H_4_ *L*)^2+^ adopt *syn* orientations. Intra­molecular N—H⋯N and N—H⋯O hydrogen bonds lead to the formation of *S*(5) motifs. In the crystal, neighbouring cations are connected by π–π inter­actions between pyridinium units with a centroid–centroid distance of 3.600 (1) Å. Moreover, the crystal components are assembled into two-dimensional layers *via* N—H⋯O and O—H⋯O hydrogen bonds, with no direct hydrogen-bonding inter­actions between cations.

## Related literature

For the use of *N*′,*N*′^2^-bis­[(1*E*)-1-(2-pyrid­yl)ethyl­idene]ethane­dihydrazide in reactions with metal ions, see: Anđelković *et al.* (2005 ▶); Kelly *et al.* (2005 ▶); Sen *et al.* (2006 ▶). For hydrogen bonds, see: Bernstein *et al.* (1995 ▶); Jeffrey *et al.* (1985 ▶).
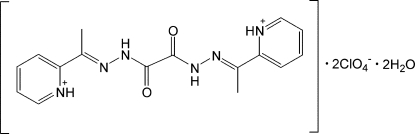


## Experimental

### 

#### Crystal data

C_16_H_18_N_6_O_2_^2+^·2ClO_4_^−^·2H_2_O
*M* *_r_* = 561.3
Monoclinic, 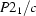

*a* = 7.0166 (3) Å
*b* = 15.6855 (5) Å
*c* = 10.1152 (4) Å
β = 90.240 (3)°
*V* = 1113.26 (7) Å^3^
*Z* = 2
Mo *K*α radiation
μ = 0.37 mm^−1^
*T* = 130 K
0.4 × 0.3 × 0.2 mm

#### Data collection

Oxford Diffraction XcaliburS CCD diffractometer
Absorption correction: multi-scan (*CrysAlis PRO*; Oxford Diffraction, 2009 ▶) *T*_min_ = 0.875, *T*_max_ = 0.929
12596 measured reflections
3402 independent reflections
2504 reflections with *I* > 2σ(*I*)
*R*_int_ = 0.037

#### Refinement

*R*[*F* ^2^ > 2σ(*F* ^2^)] = 0.043
*wR*(*F* ^2^) = 0.105
*S* = 0.98
3402 reflections
173 parameters
2 restraints
H atoms treated by a mixture of independent and constrained refinement
Δρ_max_ = 0.69 e Å^−3^
Δρ_min_ = −0.48 e Å^−3^

### 

Data collection: *CrysAlis PRO* (Oxford Diffraction, 2009 ▶); cell refinement: *CrysAlis PRO*; data reduction: *CrysAlis PRO*; program(s) used to solve structure: *SHELXS97* (Sheldrick, 2008 ▶); program(s) used to refine structure: *SHELXL97* (Sheldrick, 2008 ▶); molecular graphics: *ORTEP-3 for Windows* (Farrugia, 1997 ▶) and *Mercury* (Macrae *et al.*, 2006 ▶); software used to prepare material for publication: *SHELXL97*.

## Supplementary Material

Crystal structure: contains datablocks I, global. DOI: 10.1107/S1600536810010238/gk2261sup1.cif

Structure factors: contains datablocks I. DOI: 10.1107/S1600536810010238/gk2261Isup2.hkl

Additional supplementary materials:  crystallographic information; 3D view; checkCIF report

## Figures and Tables

**Table 1:** Hydrogen-bond geometry (Å, °)

*D*—H⋯*A*	*D*—H	H⋯*A*	*D*⋯*A*	*D*—H⋯*A*
N1—H1*N*⋯O6	0.84 (2)	1.86 (2)	2.690 (2)	168 (2)
N1—H1*N*⋯N2	0.84 (2)	2.32 (2)	2.632 (2)	102 (2)
N3—H3*N*⋯O5	0.84 (2)	2.36 (2)	3.011 (2)	134 (2)
N3—H3*N*⋯O1^i^	0.84 (2)	2.36 (2)	2.686 (2)	104 (2)
O6—H6*A*⋯O1	0.82 (2)	2.08 (2)	2.889 (2)	173 (2)
O6—H6*B*⋯O3^ii^	0.84 (2)	1.98 (2)	2.809 (2)	171 (2)

Symmetry codes: (i) 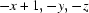; (ii) 
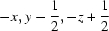
.

**Fig. 1.**
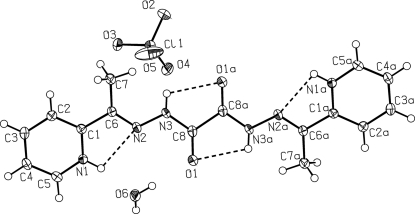
The numbering scheme in the title compound. Displacement ellipsoids are drawn at the 50% probability level. The intramolecular hydrogen bonds(N—H···O and N—H···N) are shown with dashed lines. Atoms labeled with the suffix "a" are at the symmetry position 1-x, -y, -z.

**Fig. 2.**
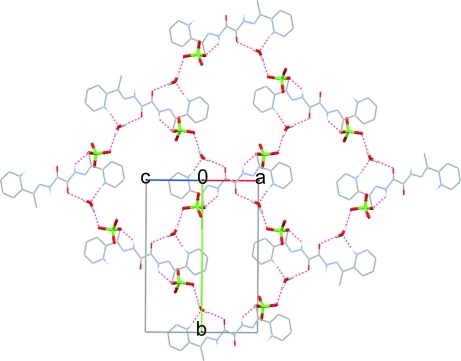
Packing diagram of the title compound showing 2D assembly parallel to (1 0 2) generated by hydrogen bonding. Hydrogen atoms, except those involved in hydrogen bonding, are omitted for clarity.

**Fig. 3.**
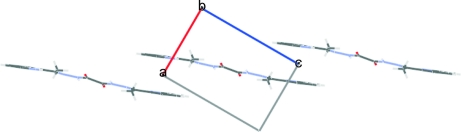
Packing diagram of the title compound showing 1D assembly parallel to [–1 0 1] generated by stacking interactions of the pyridinium fragments. View along b-axis.

## supplementary crystallographic information

### Comment

*N*',*N*'^2^-Bis[(1*E*)-1-(2-pyridyl)ethylidene]ethanedihydrazide (H_2_*L*) is usually used for the preparation of metal complexes (Anđelković *et al.* 2005; Kelly *et al.* 2005; Sen *et al.*, 2006). However, only two complexes, polynuclear complex of Cu(II) and mononuclear complex of Ni(II), with ligand H_2_*L* have been obtained and characterized so far (Sen *et al.*, 2006). These complexes have been prepared by direct reaction of *M*(AcO)_2_ [*M* = Cu(II) and Ni(II)] with H_2_*L* in 2-propanol/H_2_O (Sen *et al.*, 2006). However, in the reactions of H_2_*L* with Cu(NO_3_)_2_.3H_2_O or Cu(ClO_4_)_2_.6H_2_O in MeOH/H_2_O, hydrolysis at the hydrazide moiety occurred affording the formation of the binuclear Cu(II) complex with 2-acetylpyridine hydrazone in which oxalate ion serves as a bridge between two metal centers (Kelly *et al.*, 2005). Similarly, hydrolysis of H_2_*L* took place in the reaction with Fe(ClO_4_)_3_.6H_2_O in water, with simultaneous reduction of Fe(III) to Fe(II) by oxalic fragment affording formation of mononuclear Fe(II) complex with 2-acetylpyridine hydrazone (Anđelković *et al.*, 2005). The cited studies show that direct synthetic reactions of metal ions with the ligand H_2_*L* may be very intricate and often lead to accidental products. The title salt, (I), was obtained unintentionally as a major product in direct reaction of Zn(ClO_4_)_2_.6H_2_O with the ligand *N*',*N*'^2^-bis[(1*E*)-1-(2-pyridyl)ethylidene]ethanedihydrazide (H_2_*L*). The cation (H_4_*L*)^2+^ lies at the center of inversion at 1/2, 0, 0. The numbering scheme of (I) is given in Fig. 1. The C8—O1 bond distance of 1.214 (2) Å is consistent with the carbon–oxygen double bonding. The N3—C8 [1.352 (2) Å] and N2—C6 [1.289 (2) Å] bond distances indicate single and double CN bonding, respectively . The cation deviates from planarity. The distance between the mean plane defined by C1-C6,C8,N1-N3 atoms and that defined by respective symmetry related atoms is 0.223 Å. The structure of (I) is stabilized by intramolecular and intermolecular hydrogen bonds and their geometrical details are listed in Table 1. The *s-trans* conformation of the cation is stabilized by N–H···O hydrogen bonds (Fig. 1, Table 1). The torsion angle O1—C8—C8a—O1a [atoms labeled with the suffix "a" are at symmetry position 1–*x*, –*y*, –*z*] is 180°. The *syn* orientations of the pyridiniumimine fragments are stabilized by the N—H···N intramolecular hydrogen bonds. The torsion angle N1—C1—C6—N2 is 3.3 (2)°. The intramolecular hydrogen bonds (N–H···O and N—H···N) lead to formation of S(5) motifs (Fig. 1.) (Bernstein *et al.*, 1995). In the crystal structure all residues participate in the intermolecular hydrogen bonding (Fig. 2.). Solvent water molecule acts as a double donor [to O1 and O3 at –*x*, *y* – 1/2, –*z* + 1/2] and a single acceptor. The pyridinium and hydrazone nitrogens serve as double hydrogen bond donors with one component intra and the other intermolecular. As suggested by Jeffrey *et al.*, 1985, this type of H-bond is called three-center. Perchlorate groups and water molecules mediate in joining together the cation molecules (Fig. 2.). Each cation is H-bonded to two perchlorate groups and two water molecules. The oxygen atoms (O3 and O5) from perchlorate group serve as H-bond acceptors. The O5 accepts hydrogen from hydrazone nitrogen and O3 from water molecule. The other hydrogen from water molecule is being donated to carbonyl oxygen (O1) of cation molecule. This system of H-bond interactions spreads in two-dimensions parallel to (1 0 2). The heteroaromatic rings of the neighbouring cations are involved in π–π interactions (Fig. 3). The aromatic rings are found to be parallel-displaced. Namely, the distance between the centers of gravity of aromatic rings (C1—C5,N1) and (C1b—C5b,N1b) [atoms labeled with the suffix "b" are at symmetry position –*x*, –*y*, 1–*z*] is 3.600 (1) Å with the center of gravity displaced distance of 1.502 Å. Cation molecules connected by π–π interactions between pyridinium units extend in a stairs-like manner along [101].

### Experimental

Zn(ClO_4_)_2_.6H_2_O (0.32 g, 0.85 mmol) and H_2_*L* (0.27 g, 0.85 mmol) were suspended in MeOH (30 cm^3^). To the light yellow suspension 4–5 drops of HClO_4_ were added and the resulting yellow solution was refluxed for 1 h at 338 K. Upon cooling to room temperature and filtration, a light yellow microcrystalline product was obtained. Yield: 56%; mp. 511–513 K; molar conductivity (DMF, 1.10 ^–3^ mol dm^–3^) λ_M_ = 160 Ω^–1^cm^2^ mol^–1^. Solubility: insoluble in water and ethanol, soluble in acetonitrile and dimethylsulfoxide. The molar conductivity of a DMF solution of the ligand salt (1.10 ^–3^ mol dm^–3^) was measured at room temperature on a Jenway-4009 digital conductivity meter.

### Refinement

The H atoms connected to C atoms were positioned geometrically (C—H = 0.95 - 0.98 Å) and treated as riding on their carrier atoms with *U*_iso_(H) = 1.2U_eq_(C). H atoms at nitrogen were found in electron-density difference maps and refined freely. In order to adjust distances of hydrogen atoms of water molecule DFIX instruction was used with the target value of 0.84 (2) Å (O6—H). The crystal was pseudomerohedrally twinned with the twin law (1 0 0 0 -1 0 0 0 -1) in the reciprocal space. The refinement gave with the 6 % content of the minor component.

### Crystal data

**Table:**

C_16_H_18_N_6_O_2_^2+^·2ClO_4_^−^·2H_2_O	*F*(000) = 580
*M**_r_* = 561.3	*D*_x_ = 1.674 Mg m^−^^3^
Monoclinic, *P*2_1_/*c*	Mo *K*α radiation, λ = 0.71073 Å
Hall symbol: -P 2ybc	Cell parameters from 4422 reflections
*a* = 7.0166 (3) Å	θ = 2.9–32.2°
*b* = 15.6855 (5) Å	µ = 0.37 mm^−^^1^
*c* = 10.1152 (4) Å	*T* = 130 K
β = 90.240 (3)°	Plate, colourless
*V* = 1113.26 (7) Å^3^	0.4 × 0.3 × 0.2 mm
*Z* = 2	

### Data collection

**Table:**

Oxford Diffraction XcaliburS CCD diffractometer	3402 independent reflections
graphite	2504 reflections with *I* > 2σ(*I*)
Detector resolution: 16.356 pixels mm^-1^	*R*_int_ = 0.037
ω scans and φ scans	θ_max_ = 30.5°, θ_min_ = 2.9°
Absorption correction: multi-scan (*CrysAlis PRO*; Oxford Diffraction, 2009)	*h* = −9→10
*T*_min_ = 0.875, *T*_max_ = 0.929	*k* = −22→20
12596 measured reflections	*l* = −12→14

### Refinement

**Table:**

Refinement on *F*^2^	Primary atom site location: structure-invariant direct methods
Least-squares matrix: full	Secondary atom site location: difference Fourier map
*R*[*F*^2^ > 2σ(*F*^2^)] = 0.043	Hydrogen site location: inferred from neighbouring sites
*wR*(*F*^2^) = 0.105	H atoms treated by a mixture of independent and constrained refinement
*S* = 0.98	*w* = 1/[σ^2^(*F*_o_^2^) + (0.0575*P*)^2^] where *P* = (*F*_o_^2^ + 2*F*_c_^2^)/3
3402 reflections	(Δ/σ)_max_ < 0.001
173 parameters	Δρ_max_ = 0.69 e Å^−^^3^
2 restraints	Δρ_min_ = −0.48 e Å^−^^3^

### Special details

**Table:**

Geometry. All esds (except the esd in the dihedral angle between two l.s. planes) are estimated using the full covariance matrix. The cell esds are taken into account individually in the estimation of esds in distances, angles and torsion angles; correlations between esds in cell parameters are only used when they are defined by crystal symmetry. An approximate (isotropic) treatment of cell esds is used for estimating esds involving l.s. planes.
Refinement. Refinement of F^2^ against ALL reflections. The weighted R-factor wR and goodness of fit S are based on F^2^, conventional R-factors R are based on F, with F set to zero for negative F^2^. The threshold expression of F^2^ > 2sigma(F^2^) is used only for calculating R-factors(gt) etc. and is not relevant to the choice of reflections for refinement. R-factors based on F^2^ are statistically about twice as large as those based on F, and R- factors based on ALL data will be even larger.

### Fractional atomic coordinates and isotropic or equivalent isotropic displacement parameters (Å^2^)

**Table:**

	*x*	*y*	*z*	*U*_iso_*/*U*_eq_	
Cl1	−0.06740 (7)	0.17109 (3)	0.03091 (5)	0.02258 (12)	
O1	0.5097 (2)	−0.08816 (8)	0.10597 (12)	0.0239 (3)	
O2	−0.0958 (2)	0.23504 (9)	−0.06811 (15)	0.0357 (4)	
O3	−0.2032 (3)	0.18404 (10)	0.13518 (17)	0.0553 (6)	
O4	−0.0935 (2)	0.08764 (8)	−0.02205 (14)	0.0331 (3)	
O5	0.1188 (3)	0.17823 (11)	0.0853 (3)	0.0705 (7)	
O6	0.3496 (2)	−0.14953 (9)	0.35088 (15)	0.0285 (3)	
N1	0.2466 (2)	−0.01871 (9)	0.50693 (15)	0.0167 (3)	
N2	0.3686 (2)	0.03179 (9)	0.27403 (15)	0.0190 (2)	
N3	0.4253 (2)	0.05006 (10)	0.14877 (14)	0.0190 (2)	
C1	0.2678 (2)	0.06619 (11)	0.48485 (17)	0.0168 (3)	
C2	0.2139 (3)	0.12184 (11)	0.58310 (18)	0.0206 (4)	
H2	0.2278	0.1815	0.5705	0.025*	
C3	0.1395 (3)	0.09094 (12)	0.70044 (18)	0.0226 (4)	
H3	0.1008	0.1295	0.7675	0.027*	
C4	0.1214 (2)	0.00423 (12)	0.72004 (18)	0.0214 (4)	
H4	0.0714	−0.0176	0.8004	0.026*	
C5	0.1775 (2)	−0.05004 (11)	0.62031 (17)	0.0193 (4)	
H5	0.1672	−0.11	0.632	0.023*	
C6	0.3414 (2)	0.09391 (11)	0.35522 (17)	0.0174 (3)	
C7	0.3775 (3)	0.18669 (11)	0.3335 (2)	0.0271 (4)	
H7A	0.4424	0.1947	0.2488	0.041*	
H7B	0.4579	0.2088	0.4052	0.041*	
H7C	0.2559	0.2174	0.3323	0.041*	
C8	0.4826 (2)	−0.01508 (11)	0.07098 (18)	0.0188 (3)	
H1N	0.279 (3)	−0.0545 (14)	0.449 (2)	0.026 (4)*	
H3N	0.401 (3)	0.0985 (14)	0.118 (2)	0.026 (4)*	
H6A	0.388 (4)	−0.1348 (17)	0.2784 (19)	0.049 (6)*	
H6B	0.295 (4)	−0.1971 (13)	0.351 (3)	0.049 (6)*	

### Atomic displacement parameters (Å^2^)

**Table:**

	*U*^11^	*U*^22^	*U*^33^	*U*^12^	*U*^13^	*U*^23^
Cl1	0.0305 (2)	0.01659 (19)	0.0206 (2)	0.00294 (16)	0.00279 (17)	0.00130 (16)
O1	0.0335 (7)	0.0212 (6)	0.0172 (7)	0.0020 (5)	0.0033 (5)	0.0015 (5)
O2	0.0530 (10)	0.0281 (7)	0.0262 (8)	0.0080 (7)	0.0064 (7)	0.0114 (6)
O3	0.1039 (16)	0.0265 (8)	0.0358 (10)	0.0169 (9)	0.0408 (10)	0.0046 (7)
O4	0.0494 (9)	0.0214 (7)	0.0285 (8)	−0.0010 (6)	0.0064 (7)	−0.0057 (6)
O5	0.0498 (12)	0.0292 (9)	0.132 (2)	−0.0002 (8)	−0.0469 (13)	0.0052 (10)
O6	0.0401 (8)	0.0207 (7)	0.0247 (8)	−0.0045 (6)	0.0116 (6)	−0.0010 (6)
N1	0.0174 (7)	0.0175 (7)	0.0153 (7)	0.0006 (5)	0.0009 (6)	−0.0004 (5)
N2	0.0242 (6)	0.0206 (5)	0.0121 (5)	0.0002 (4)	0.0031 (4)	0.0024 (4)
N3	0.0242 (6)	0.0206 (5)	0.0121 (5)	0.0002 (4)	0.0031 (4)	0.0024 (4)
C1	0.0158 (8)	0.0182 (8)	0.0163 (8)	0.0001 (6)	0.0001 (6)	0.0010 (6)
C2	0.0240 (9)	0.0183 (8)	0.0195 (9)	0.0002 (6)	0.0019 (7)	−0.0013 (6)
C3	0.0258 (9)	0.0258 (9)	0.0161 (9)	0.0000 (7)	0.0011 (7)	−0.0035 (7)
C4	0.0222 (9)	0.0274 (9)	0.0145 (8)	−0.0003 (7)	0.0016 (7)	0.0035 (7)
C5	0.0193 (9)	0.0207 (8)	0.0180 (9)	−0.0016 (6)	−0.0011 (7)	0.0038 (7)
C6	0.0170 (8)	0.0195 (8)	0.0156 (8)	−0.0005 (6)	0.0019 (6)	0.0004 (6)
C7	0.0410 (12)	0.0193 (9)	0.0209 (10)	−0.0028 (8)	0.0097 (8)	0.0014 (7)
C8	0.0192 (8)	0.0219 (8)	0.0151 (8)	−0.0031 (6)	0.0012 (6)	−0.0016 (7)

### Geometric parameters (Å, °)

**Table:**

Cl1—O5	1.4198 (17)	C1—C2	1.377 (2)
Cl1—O4	1.4259 (14)	C1—C6	1.477 (2)
Cl1—O2	1.4309 (14)	C2—C3	1.386 (3)
Cl1—O3	1.4386 (17)	C2—H2	0.95
O1—C8	1.214 (2)	C3—C4	1.381 (3)
O6—H6A	0.815 (17)	C3—H3	0.95
O6—H6B	0.839 (17)	C4—C5	1.379 (3)
N1—C5	1.340 (2)	C4—H4	0.95
N1—C1	1.359 (2)	C5—H5	0.95
N1—H1N	0.84 (2)	C6—C7	1.493 (2)
N2—C6	1.289 (2)	C7—H7A	0.98
N2—N3	1.360 (2)	C7—H7B	0.98
N3—C8	1.352 (2)	C7—H7C	0.98
N3—H3N	0.84 (2)	C8—C8^i^	1.532 (4)
			
O5—Cl1—O4	109.55 (10)	C4—C3—H3	119.9
O5—Cl1—O2	109.96 (11)	C2—C3—H3	119.9
O4—Cl1—O2	111.30 (9)	C5—C4—C3	118.46 (17)
O5—Cl1—O3	108.40 (14)	C5—C4—H4	120.8
O4—Cl1—O3	108.69 (10)	C3—C4—H4	120.8
O2—Cl1—O3	108.87 (9)	N1—C5—C4	120.35 (16)
H6A—O6—H6B	114 (3)	N1—C5—H5	119.8
C5—N1—C1	122.70 (15)	C4—C5—H5	119.8
C5—N1—H1N	116.7 (15)	N2—C6—C1	113.35 (15)
C1—N1—H1N	120.6 (15)	N2—C6—C7	128.13 (17)
C6—N2—N3	118.61 (14)	C1—C6—C7	118.52 (15)
C8—N3—N2	118.15 (15)	C6—C7—H7A	109.5
C8—N3—H3N	122.0 (14)	C6—C7—H7B	109.5
N2—N3—H3N	118.4 (15)	H7A—C7—H7B	109.5
N1—C1—C2	118.18 (16)	C6—C7—H7C	109.5
N1—C1—C6	118.26 (15)	H7A—C7—H7C	109.5
C2—C1—C6	123.53 (16)	H7B—C7—H7C	109.5
C1—C2—C3	120.13 (17)	O1—C8—N3	126.18 (17)
C1—C2—H2	119.9	O1—C8—C8^i^	122.6 (2)
C3—C2—H2	119.9	N3—C8—C8^i^	111.16 (18)
C4—C3—C2	120.17 (17)		
			
C6—N2—N3—C8	169.62 (16)	N3—N2—C6—C1	176.07 (14)
C5—N1—C1—C2	−0.7 (2)	N3—N2—C6—C7	−4.8 (3)
C5—N1—C1—C6	−178.72 (16)	N1—C1—C6—N2	3.3 (2)
N1—C1—C2—C3	−0.3 (3)	C2—C1—C6—N2	−174.65 (17)
C6—C1—C2—C3	177.63 (16)	N1—C1—C6—C7	−175.92 (16)
C1—C2—C3—C4	0.9 (3)	C2—C1—C6—C7	6.1 (3)
C2—C3—C4—C5	−0.5 (3)	N2—N3—C8—O1	−9.0 (3)
C1—N1—C5—C4	1.1 (3)	N2—N3—C8—C8^i^	172.93 (16)
C3—C4—C5—N1	−0.5 (3)		

Symmetry codes: (i) −*x*+1, −*y*, −*z*.

### Hydrogen-bond geometry (Å, °)

**Table:**

*D*—H···*A*	*D*—H	H···*A*	*D*···*A*	*D*—H···*A*
N1—H1N···O6	0.84 (2)	1.86 (2)	2.690 (2)	168 (2)
N1—H1N···N2	0.84 (2)	2.32 (2)	2.632 (2)	102 (2)
N3—H3N···O5	0.84 (2)	2.36 (2)	3.011 (2)	134 (2)
N3—H3N···O1^i^	0.84 (2)	2.36 (2)	2.686 (2)	104 (2)
O6—H6A···O1	0.82 (2)	2.08 (2)	2.889 (2)	173 (2)
O6—H6A···N2	0.82 (2)	2.62 (3)	2.952 (2)	106 (2)
O6—H6B···O3^ii^	0.84 (2)	1.98 (2)	2.809 (2)	171 (2)
C2—H2···O5^iii^	0.95	2.33	3.206 (2)	152
C4—H4···O4^iv^	0.95	2.50	3.384 (2)	155
C5—H5···O2^ii^	0.95	2.56	3.460 (2)	157
C7—H7A···N3	0.98	2.49	2.864 (2)	103

Symmetry codes: (i) −*x*+1, −*y*, −*z*; (ii) −*x*, *y*−1/2, −*z*+1/2; (iii) ; (iv) −*x*, −*y*, −*z*+1.
